# LAG3, TIM3 and TIGIT: New Targets for Immunotherapy and Potential Associations with Radiotherapy

**DOI:** 10.3390/curroncol32040230

**Published:** 2025-04-15

**Authors:** Camil Ciprian Mireștean, Roxana Irina Iancu, Dragoș Petru Teodor Iancu

**Affiliations:** 1 Regional Institute of Oncology, 700483 Iasi, Romania; mc3313@yahoo.com; 2 “Gr. T. Popa” University of Medicine and Pharmacy, 700115 Iasi, Romania; roxana.iancu@umfiasi.ro; 3 “St. Spiridon” Emergency Universitary Hospital, 700111 Iasi, Romania

**Keywords:** radiotherapy, SRS, immunotherapy, ICI, PD-1, PD-L1, CTLA-4, TIGIT, LAG-3, TIM-3, synergy

## Abstract

The combination of immunotherapy and radiotherapy has demonstrated synergistic potential, especially when a combination of immune checkpoint inhibitors (ICIs) is administered. Cytotoxic T-Lymphocyte-Associated Protein-4 (CTLA-4) inhibitors and Programmed Death-Ligand 1 (PD-L1) inhibitors or Programmed Cell Death Protein 1 (PD-1) inhibitors have been assessed in both clinical and preclinical studies; the addition of radiotherapy activates immunomodulatory mechanisms materialized by an effect similar to “in situ” vaccination or the “abscopal” distant response of lesions outside the irradiation field. The new therapeutic targets (T cell immune-receptor with Ig and ITIM domains (TIGIT), Lymphocyte activating gene 3 (LAG-3), and T cell Ig- and mucin-domain-containing molecule-3 (TIM-3)) associated with traditional ICIs and radiotherapy open new perspectives to the concept of immuno-radiotherapy. The dynamic evaluation of T lymphocyte expression involved in the antitumor immune response, both in the tumor microenvironment (TME) and in the tumor itself, could have biomarker value in assessing the response to combination therapy with traditional and new ICIs in association with irradiation. Preclinical data justify the initiation of clinical trials in various tumor pathologies to explore this concept.

## 1. Introduction

Immune checkpoint inhibitors (ICIs) have demonstrated the ability to restore antitumor immunity, particularly by restoring, inducing, and activating cytotoxic T lymphocytes, resulting in a long-lasting antitumor immune response. In 2011, the US Food and Drug Administration (FDA) approved the first ICI, ipilimumab, a cytotoxic T-Lymphocyte-Associated Protein-4 (CTLA-4) inhibitor [1]. In 2024, Lu et al. reported the existence of seven immuno-therapeutic agents approved in clinical practice: the Anti-Programmed Cell Death Protein 1 (PD-1) inhibitors nivolumab and pembrolizumab, and the Anti- Programmed Cell Death Protein Ligand 1 (PD-L1) inhibitors atezolizumab, durvalumab, avelumab, and cemiplimab [2]. Subsequently, retifanlimab, toripalimab, and tremelimumab were reported by Jeddeo Paul et al. among the 11 ICIs approved by the FDA. Even though, in clinical practice, they are currently used as single agents, in combination with chemotherapy or in dual combination targeting CTLA-4/PD-1 or PD-L1 inhibition, the results of immunotherapy remain suboptimal in most cases [3]. The association of radiotherapy with ICI is still a controversial topic, considering the immunomodulatory mechanisms of irradiation, both of antitumor response augmentation and immunosuppression. The presentation of tumor neo-antigens and the remodeling of the immune microenvironment are mechanisms underlying this interaction. In the clinic, the “abscopal” effect of tumor response of a tumor outside the irradiation field and the reconversion of poorly immunogenic (cold) tumors into highly immunogenic or inflamed (hot) tumors by irradiation are examples of interactions of irradiation with immunotherapy [4,5,6,7,8,9].

## 2. LAG3, TIM3 and TIGIT—New Targets in “Immunotherapy Collimator”

We aim to summarize the data describing the potential interactions between radiotherapy and the novel targets T cell immune-receptor with Ig and ITIM domains (TIGIT), Lymphocyte activating gene 3 (LAG-3), T cell Ig- and mucin-domain-containing molecule-3 (TIM-3).

As a surface protein expressed on activated and memory regulatory T lymphocytes, TIGIT includes an immunoglobulin variable domain, a transmembrane domain, and a tyrosine-based immunoreceptor inhibitor. By binding to the poliovirus receptor, but also by modulating the level of cytokines produced by dendritic cells, this immunoreceptor can have an immunosuppressive effect [10]. As early as 2009, Boles and colleagues mentioned immune receptors for Nectins and Necl molecules whose expression is downregulated during tumorigenesis. The Washington University cell adhesion molecule expressed on human follicular T helper (TFH) cells binds to the poliovirus receptor, a member of the Nectin/Necl family, and produces T cell-dependent antibody responses [11]. A member of the CD28 protein family, also known as TIGIT and WUCAM, it is expressed on NK cells, activated and memory T cells, and Tregs, and Levin et al. mention their potential to block immune responses similar to that of CTLA-4 [12].

The TIGIT protein is overexpressed on immune cells, such as CD4+ and CD8+ T cells, but also natural killer (NK) cells, having an essential role in immunosuppression, especially through interaction with the tumor microenvironment (TME). Competitive binding with CD155 to the detriment of CD112 leads to the deactivation of T and NK cells. Regulatory T lymphocytes (TREGs) are stabilized by TIGIT which inhibits the PI3K/AKT/mTOR pathway [13].

LAG-3, a receptor protein belonging to the immunoglobulin superfamily, has been identified on activated T lymphocytes, playing a role in maintaining immune system homeostasis and promoting immune escape in the tumor microenvironment (TME). LAG-3 inhibitors can modulate T lymphocyte activity by associating with the canonical ligand MHC II, as well as with Galectin-3 and LSECtin, but can enhance antitumor immunity by limiting the FGL1-LAG-3 interaction [14,15,16,17,18,19].

Activation of naive CD4(+) T-helper cells results in the production of Th1 and Th2 effector cells, which produce different types of cytokines. Monney et al. report for the first time the identification of a transmembrane protein, Tim-3, expressed on differentiated Th1 cells with a role in regulating macrophage function and in the development of autoimmune diseases [20].

TIM-3 (T-cell immunoglobulin- and mucin domain-containing protein 3) is a protein expressed on various cells of the immune system, including T cells, dendritic cells, and natural killer (NK) cells. TIM-3 plays an essential role in suppressing immune responses. TIM-3 is also implicated in reducing immune activity in patients with cancer and infections. T-cell immunoglobulin- and mucin domain-containing protein 3 (TIM-3) is considered an immune checkpoint receptor due to its interaction with GAL-9, PtdSer, HMGB1, and CEACAM and is found on the surface of Th1 T helper cells, as well as monocytes, macrophages, and cytotoxic lymphocytes (CTLs), NKs, and dendritic cells (DCs). TIM-3-associated immune evasion specifically involves exhausted T cells that have lost their ability to respond to antigens [21,22,23,24].

TIGIT blockade in combination with radiotherapy has been studied in both esophageal cancer tumor models and in mouse models through activation of the cGAS-STING pathway, induction of chemokine expression, and release of nuclear high-mobility protein from the box-1 group. The study proposed by Zhao and collaborators highlighted the increase in TIGIT/CD155 expression in T cells and DCs after irradiation [25]. As a concept, the authors intended to evaluate a possible augmented benefit by combining TIGIT blockade with irradiation. The mechanism of amplification of the irradiation benefit is CD8+ T cell-dependent, with TIGIT blockade promoting tumor infiltration with DCs and activation of CD8+ T lymphocytes. In the mouse model, CD103+ DCs were required to activate the antitumor immune response, and Flt3L therapy increased the amplitude of the effect. Previously, the results of the phase II CITYSCAPE trial demonstrated the need for a therapeutic combination, as TIGIT blockade was considered insufficient to support an antitumor immune response. The TIGIT inhibitor tiragolumab (MTIG7192A, RG-6058) plus atezolizumab demonstrated a consistent response only in cases of non-small-cell lung carcinoma that involved PD-L1 expression higher than 50% [26]. For the group of patients with esophageal cancer, CD8 + T, CD4 + T, and NK cell infiltration levels in the tumor were evaluated both before and after neo-adjuvant therapy with chemo-radiotherapy. TIGIT + CD8 + T cells were strongly increased 4 weeks after neo-adjuvant therapy but decreased 8 weeks after neoadjuvant treatment. However, the authors suggest that these tumor infiltrates could also have an immunosuppressive role. Anti-TIGIT therapy alone has demonstrated favorable results only in certain melanoma cell lines, with the combination with radiotherapy being able to control tumor growth in these models [26,27].

Mice with subcutaneous CT26 colon tumors irradiated with an SARRP device in association or not with ICI were evaluated from the point of view of the response to irradiation in terms of myeloid, cytokine, and lymphoid cells and analyzed by flow cytometry, but also by RNA sequencing. Different treatment sequences were proposed; 18x2Gy and 3x8Gy were associated the highest rate of tumor growth delays compared to 1x16.4Gy [28]. The lymphoid response was associated with the use of the 3x8Gy and 1x16.4Gy irradiation sequences, and the myeloid response (myeloid-derived suppressor cells, tumor-associated macrophage 2) was associated with 18x2Gy. The 18x2Gy sequence increased PD-L1 expression, TIGIT expression was increased by 3x8Gy but decreased after irradiation with the 18x2Gy sequence. For 9 out of 10 mice, the combination of anti-TIGIT and anti-PD-L1 therapy brought a durable response, and anti-PD-L1 and RT were associated with two thirds of the complete response in the group treated with 18x2Gy. The authors note the pioneering study that associates radiotherapy and anti-TIGIT therapy [4,27].

LAG-3 could induce T-cell dysfunction and interacts with soluble fibrinogen ligand protein-1 (FGL1), LAG-3 expression being even higher than that of PD-1. A bispecific peptide LFOP targeting PD-1/PD-L1 and LAG-3/FGL1 has the ability to activate T cells and increase tumor infiltration with T lymphocytes, and the effect is synergistic with irradiation, amplifying the immune response [28,29]. As previously mentioned, MHC II is the canonical ligand of LAG-3, with LAG-3 MHC II and FGL1 binding sites being different. And in the case of LAG-3 MHC II Gal-3 and LSECtin, the situation is similar, so inhibition of one axis does not affect other interactions [30,31].

Microwave and nanoparticle ablation was tested in an MC38 mouse model used for preclinical evaluation of colon cancer, associated with anti-LAG-3 therapy [32]. The combined methods led to a significant tumor response by both increasing survival and reducing tumor growth rate. The lung cancer model treated with NBTXR3 metal nanoparticles plus microwave irradiation, anti-PD-1, anti-TIGIT and anti-LAG-3 demonstrated increased efficacy compared to a protocol that excluded LAG-3 blockade. Also, CD8+ IFNγ+ TNFα+ TILs increased in the case of the addition of LAG-3, with LAG-3 blockade amplifying the immune response mediated by CD8+ T lymphocytes [32,33].

The synergistic potential between PD-1 inhibition and LAG-3 inhibition could increase the response rate to immunotherapy in gastroesophageal cancer. A phase Ib study evaluated neoadjuvant nivolumab or nivolumab-relatlimab in combination with chemo-radiotherapy, with the study group including 32 patients with resectable stage II or stage III gastroesophageal cancer. The study evaluated safety, molecular, functional, and immune response, pathological complete response (pCR) and major pathological response (MPR), recurrence-free survival (RFS) and overall survival (OS). The study cohort that combined nivolumab and relatinib required a dose adjustment to obtain a favorable safety profile, and the pCRs were 40% and 21.4% with an MPR rate of 53% and 57.1% for nivolumab and nivolumab-relatinib plus chemo-radiotherapy, respectively. PD-1 and LAG-3 expression, as well as circulating tumor DNA (ctDNA) expression pre- and postoperatively, was a prognostic factor. The study demonstrates the feasibility of dual immune therapy followed by neo-adjuvant chemo-radiotherapy [34,35,36,37].

Radiotherapy combined with NBTXR3 plus simultaneous blockade of three immune checkpoint receptors, PD1, LAG3 and TIGIT, was evaluated in an animal model of lung cancer resistant to anti-PD1 therapy. Immunotherapy was administered intraperitoneally and irradiation at a dose of 12Gy was performed on days 8, 9 and 10 at the level of primary tumors. The association of a triple immune blockade with nanoparticles with or without irradiation was associated with tumor control rates of 30%, but also generated a transcriptional signature of native and innate immunity, the immune memory effect being also highlighted in cases where tumor re-engraftment was prevented [38].

Peng et al. aimed to evaluate the expression and response of PD-1, TIM-3, LAG-3 after neo-adjuvant radiotherapy in rectal cancer and to evaluate the optimal treatment protocol with combined immunotherapy [39]. For 13 cases, the expression of PD-1, TIM-3, LAG-3, CD8 and CD3 was evaluated before neoadjuvant treatment, and for 76 cases, the immune–histochemical expression of the same previously mentioned variables was evaluated. Immunohistochemistry was performed to detect the expression of PD-1, TIM-3, LAG-3, CD8 and CD3. The expression of these molecules was detected in specimens from 76 patients with rectal cancer after neo-adjuvant radiotherapy and 13 of these patients before NRT. Neo-adjuvant therapy increased the expression of PD-1 and immune cells (ICs) LAG-3 from 0 to 3% and from 5% to 45%, respectively. TIM-3 expression in immune cells and tumor cells was, however, downregulated. The authors also noted higher LAG-3 expression (22.5% vs. 8%) in cases treated with short-term irradiation. PD-1, ICs TIM-3, ICs LAG-3, CD3, and CD8 were correlated with disease-free survival (DFS). PD-1 and ICs TIM-3 increased after neo-adjuvant treatment, supporting the possible benefit of combined immunotherapy associated with radio-chemotherapy in rectal cancer [39].

Diffuse midline gliomas with H3-K27M mutation are brain tumors, with the therapy being currently disappointing. Aptamers are short single-stranded oligonucleotides that are able to bind with high affinity and specificity with certain molecules. The use of a TIM-3 aptamer (TIM-3 Apt) demonstrated the possibility of obtaining an immune infiltration generating a specific response. The effect was amplified by the addition of radiotherapy, the results being confirmed in two murine models and the benefit being materialized in an increase in survival. Also, the combined treatment increased the proportion of myeloid cell populations and the CD8-to-Treg ratio in TME. Based on these results, the authors propose the initiation tumor of phase I studies to exploit this association between TIM-3 Apt and radiotherapy for the treatment of diffuse midline gliomas with H3-K27M mutation [40,41].

The mechanisms of resistance to radiotherapy and RT and to PD-L1 blockade were evaluated in orthotopic tumors of murine head and neck squamous cell carcinoma (HNSCC), using anti-PD-L1, anti-TIM-3 blockade with or without radiotherapy. The results of the study revealed the overexpression of TIM-3 expression in CD8 T cells and Tregs in cases exposed to anti-PD-L1 immunotherapy and irradiation. Even though the addition of anti-TIM-3 immunotherapy led to a significant response, this was not durable. During the response period, increased T cell cytotoxicity and decreased Treg expression were noted. Treg reappearance was associated with therapeutic failure. Analyzing the results, the authors mention the essential role of Treg inhibition in obtaining a durable response to immunotherapy associated with radiotherapy [42,43].

The evaluation of the effects of dual PD-1 and Tim-3 blockade associated with irradiation in human glioblastoma multiforme was the basis of a preclinical study that included C57BL/6 mice implanted with the murine glioma cell line GL261-luc2. Randomization was proposed in eight arms including a control group and groups to which stereotactic radiosurgery (SRS) and anti-PD-1 and anti-TIM-3 immunotherapy were added, both in mono-immunotherapy and in dual association. Neither SRS nor anti-TIM-3 immunotherapy brought benefit in OS, but immunotherapy with anti-PD1 prolonged the median OS by 33 days. The association of anti-PD-1 therapy with anti-TIM-3 brought a 100-day benefit in median OS compared to the control group. The authors mention the superiority of dual immunotherapy by blocking the Tim-3 and PD-1 pathways compared to any single immune therapy [44].

## 3. Anti-TIM-3 Plus Anti-PD-1 and Radiotherapy—First Steps in Clinical Trials

A phase I study evaluates the side effects of SRS in combination with dual immune blockade with MBG453 and spartalizumab in recurrent glioblastoma multiforme. Cases of glioblastoma or grade IV gliosarcoma with gross tumor volume (GTV) ≤ 5 cm were accepted into the study. In general, the study includes cases treated multimodally with surgery, radiotherapy and chemotherapy with temozolomide, with the exception of cases with a known unmethylated MGMT promoter. A maximum of two relapses confirmed by biopsy or contrast-enhanced nuclear magnetic resonance imaging (MRI) is accepted into the study. The NCT03961971 study opens the horizons in clinical research towards the implementation of these associations of new immune target therapies with classical immunotherapy and radiotherapy [45].

## 4. Conclusions

The synergistic effects of immunotherapy and radiotherapy have already been demonstrated in preclinical and clinical studies including PD-1/PD-L1 and CTLA-4 inhibitors. Even if the benefit/detriment ratio still remains a subject of debate, due to immunomodulatory mechanisms that may involve both augmentation of the antitumor immune response and the generation of an immunosuppressive TME, preclinical data involving new immune targets, LAG-3, TIGIT and TIM-3, justify the initiation of clinical trials involving both traditional immunotherapies and inhibitors of these targets in combination with radiotherapy. The dynamic evaluation of the expression of T lymphocytes involved in the antitumor immune response both from the TME and from the tumor itself could provide these variables with biomarker value in the evaluation of the response to combined therapy with traditional and new ICIs in combination with irradiation.
